# The relation between childhood adversity and adult obesity in a population-based study in women and men

**DOI:** 10.1038/s41598-021-93242-4

**Published:** 2021-07-07

**Authors:** Toni Fleischer, Christine Ulke, Manfred Beutel, Harald Binder, Elmar Brähler, Hamimatunnisa Johar, Seryan Atasoy, Johannes Kruse, Daniëlle Otten, Ana N. Tibubos, Daniela Zöller, Sven Speerforck, Hans J. Grabe, Karl-Heinz Ladwig, Georg Schomerus

**Affiliations:** 1 Department of Psychiatry and Psychotherapy, University Medicine Greifswald, Greifswald, Germany; 2 Department of Psychiatry and Psychotherapy, University of Leipzig Medical Center, Semmelweisstrasse 10, 04103 Leipzig, Germany; 3 Department of Psychosomatic Medicine and Psychotherapy, University Medical Center, Johannes Gutenberg-University Mainz, Mainz, Germany; 4 Institute of Medical Biometry and Statistics, Faculty of Medicine and Medical Center, University of Freiburg, Freiburg, Germany; 5 Institute of Epidemiology, Mental Health Research Unit, Helmholtz Zentrum München, German Research Center for Environmental Health, Neuherberg, Germany; 6 Department of Psychosomatic Medicine and Psychotherapy, Klinikum Rechts Der Isar, Technische Universität München, Munich, Germany; 7 Department of Psychosomatic Medicine and Psychotherapy, University of Gießen and Marburg, Gießen, Germany

**Keywords:** Epidemiology, Public health, Weight management

## Abstract

Childhood maltreatment has been shown to relate to adult obesity. In this epidemiological study, we investigate the association between childhood maltreatment and waist-to-height-ratio (WHtR) in a sample of the German adult population, comprising of N = 2936 participants. WHtR, an indicator for risk of obesity, was the primary outcome. Childhood maltreatment was assessed by the Childhood Trauma Screener (CTS), which assesses emotional and physical neglect, abuse as well as sexual abuse. Cohort-data were harmonized and analyzed within DataSHIELD. We used multivariable regression models to estimate the association of childhood maltreatment and WHtR at different levels of adjustments for potential confounders. Overall childhood maltreatment was associated with a higher WHtR in both sexes (women: p = 0.004, men: p < 0.001); associations were no longer significant in women after adding socioeconomic variables, but remained significant in men (p = 0.013). Additionally, we were able to identify sex specific patterns for childhood maltreatment predicting the WHtR. Emotional neglect and abuse had stronger impacts on the WHtR in women than in men, whereas physical neglect and abuse had stronger impacts in men. To our knowledge, this is the first comprehensive population-based study testing various types of childhood maltreatment with WHtR in sex-, region- and weight-stratified analyses. Future studies in clinical populations are warranted to examine U-shaped correlations between increased WHtR and childhood maltreatment.

## Introduction

Obesity is a global epidemic with rising prevalence^1^, and it is associated with increased mortality^2^. The underlying mechanisms leading to adult obesity are not fully understood. Considering the rising trend in the global prevalence of obesity, the need to identify causal factors is of high priority. Childhood adversity is one potential factor that has been identified in the causal chain leading to obesity. Both conditions lead to increased risk of mental disorders^3–5^ and have been associated with brain structural changes^6,7^. Although there is evidence that early life stress may play a role in its development^8^, there is still some controversy concerning the role of childhood maltreatment.

Converging evidence has indicated that being subjected to childhood maltreatment increases the risk of developing obesity as an adult^9–15^, but some studies found no or only modest associations^16,17^. Research shows that there are considerable differences in prevalence rates of both obesity and childhood maltreatment across groups and geographical regions^18–22^ possibly due to varying methodological factors or varying definitions, which may in part explain these inconsistent findings. Furthermore, evidence of a dose–response association between trauma severity and obesity risk has been reported in a systematic review and meta-analysis, wherein a more severe childhood adversity was linked to increased obesity in adulthood^12,23^. In addition, associations between childhood maltreatment and obesity could be gender-specific. Indeed, there is some evidence that the effects of childhood maltreatment on weight varies across gender^24,25^, and gender-specific effects have been described^26–29^. However, the majority of studies were not stratified by gender, warranting further gender-specific research.

In the current study, we sought to examine the link between childhood maltreatment and body shape in adulthood, yet in sex-, weight- and region-stratified analyses by using a self-rated questionnaire that allows the evaluation of several dimensions of childhood maltreatment. The study was part of a multi-cohort consortium project (*GEnder-Sensitive Analyses of mental health;* GESA;^30^, a project dedicated to differentiate the prevalence, trajectories and risks factors of adverse psychosocial outcomes between men and women^30^. For this purpose, data from two ongoing population based longitudinal cohorts (*Study of Health in Pomerania*, SHIP; *Cooperative Health Research in the Augsburg Region, KORA*) based in two different regions across Germany were combined and analyzed within DataSHIELD^31,32^ which allows for joint analyses of cohorts without pooling individual level data, to main data protection.

The aim of this study was to examine whether childhood maltreatment, including physical and emotional childhood abuse and neglect (assessed by the Childhood Trauma Screener; CTS;^33^) predicts the waist-to-height-ratio (WHtR) through the use of population-based samples from two different regions in Germany in sex-stratified analyses. We hypothesize that overall childhood maltreatment, as assessed by CTS sum score, is a predictor of an increased WHtR in women and men. In exploratory analyses, we examine the predictive value of the different types of childhood maltreatment for body shape in sex-, region- and weight-stratified analyses.

## Results

### Sample descriptions

In either cohort, slightly more women than men were observed; the proportions in the cohorts were very similar with 52.4% women in KORA and 55.9% women in SHIP. The 5%-percentile minimal age was 35, mean and median age were around 50, with slightly older individuals in SHIP (+ 1–3 years). The WHtR was similar in both cohorts too, with only small differences in men. Current depression was more prevalent in women than in men and more frequent in KORA than in SHIP (Table 1).

**Table 1:** Descriptive analysis of the pooled data and the individual cohorts.

	Total		KORA (F4)		SHIP2/Leg	
	Women	Men	Women	Men	Women	Men
n	1578	1358	944	858	634	500
**Age**						
0.05	35	35	35	35	35	35
Mean	50	51	50	50	51	52
Median	51	51	51	50	51	53
0.95	66	66	66	66	66	66
**Education years**						
0.05	8.8	10	8	10	10	10
Mean	12.1	12.5	11.8	12.5	12.5	12.4
Median	11.2	11.6	11	12	11.5	11
0.95	17	17	17	17	17	17
**Occupation**						
> 34 h	507	965	227	640	280	325
%	32.2	71.1	24.1	74.6	44.2	65.1
15 – 34 h	429	41	309	23	120	18
%	27.2	3.02	32.7	2.7	19.0	3.6
< 15 h	124	22	110	13	14	9
%	7.86	1.62	11.7	1.5	2.2	1.8
unemployed	517	329	298	182	219	147
%	32.78	24.24	31.6	21.2	34.6	29.5
**WHtR**						
0.05	0.41	0.46	0.41	0.50	0.41	0.45
Mean	0.52	0.55	0.52	0.56	0.52	0.55
Median	0.51	0.54	0.51	0.55	0.51	0.54
0.95	0.67	0.67	0.67	0.68	0.67	0.66
**Current depression**						
n	116	39	59	21	57	18
%	7.35	2.87	6.3	2.5	9.0	3.6
**CTS sum**						
0.05	5.0	5.0	5.0	5.0	5.0	5.0
Mean	7.7	7.8	8.2	8.3	7.11	7.0
Median	6.6	7.3	7.0	8.0	6.0	6.0
0.95	13.6	12.6	14.0	13.0	13.0	12.0

WHtR = Waist-to-Height-Ratio, an index between 0 and 1 (optimal 0.5). CTS = Childhood Trauma Screener sum: sum of all five childhood trauma categories (each with 5 levels). Depression was defined by the PHQ-9 = Patient Health Questionnaire (KORA) and BDI-2 = Beck Depression Inventory-II (SHIP). Sum scores were taken and binarised none-mild vs moderate to severe depression. For further information see methods.

The strongest between-cohort dissimilarities were observed in educational years and occupation. Men, in both cohorts, as well as women in SHIP, had one educational year more than women in the KORA cohort. The occupation status in men was similar between the cohorts with men mostly working more than 35 h/week (KORA: 74.6%, SHIP: 65.1%). Unemployment was higher in men than in women, and in SHIP higher than in KORA. Women of the SHIP cohort worked more often full-time than women in KORA (Table 1).

We deemed the overall similarity of both regional samples sufficient to allow pooling the data for all further analyses.

**Figure 1:**
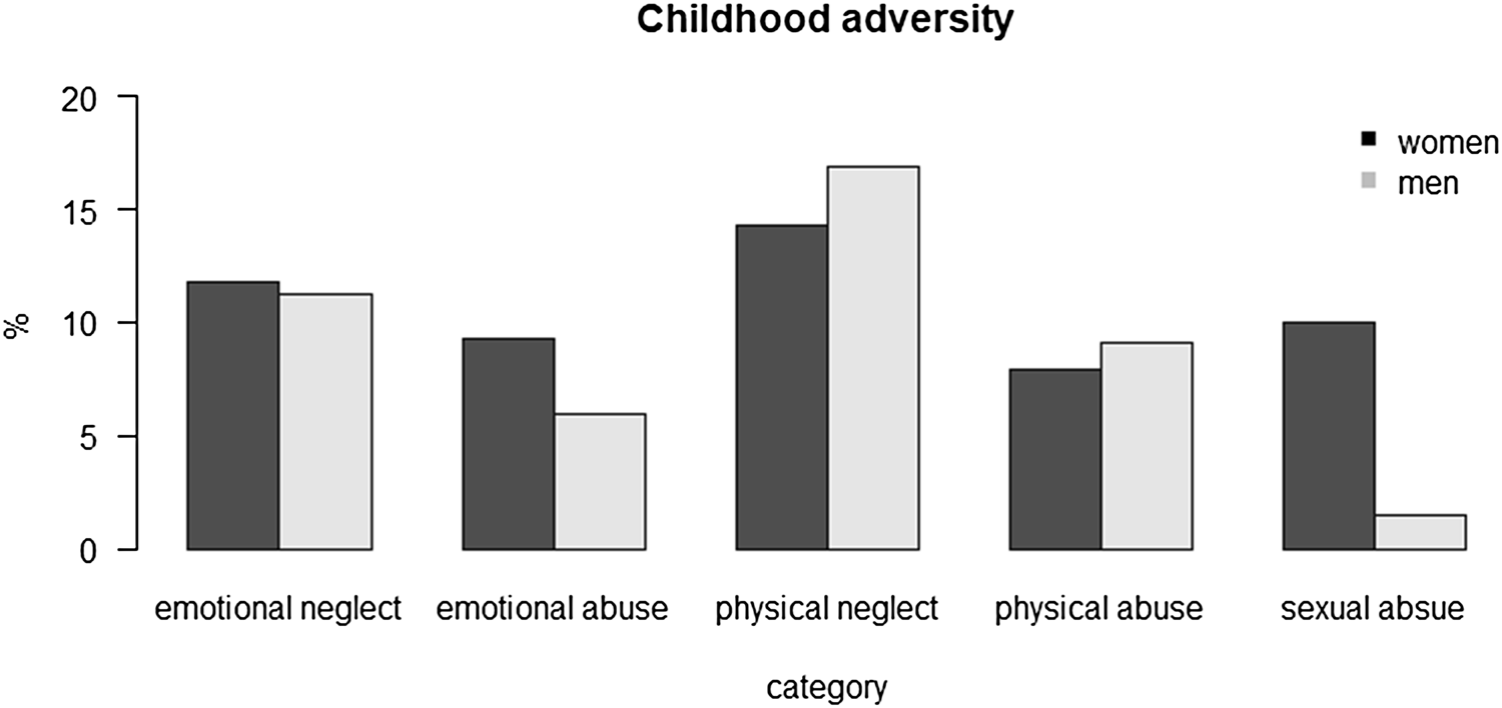
Prevalence of child maltreatment in five categories. Emotional categories as well as sexual abuse were more often reported by women, the physical categories were more often reported by men. Figure was created in R 3.5.2.

Figure 1 shows the distribution of the different types of childhood maltreatment after the binary transformation. We observed different prevalences in men and women. Emotional neglect, abuse and sexual abuse were more often reported in women, while physical neglect and abuse were more prevalent in men.

### Main analysis: CTS sum score as a predictor of WHtR

*Baseline* In multivariable regression models, the CTS sum score was significantly associated with WHtR in women (beta = 0.074, p = 0.004) and men (beta = 0.133, p < 0.001), please cf. Table 2 for the models used, and Table 3 for the results. Adding socioeconomic variables to the model, age became the strongest factor related to an increase in WHtR. Furthermore, the effect of the CTS sum score on WHtR remained significant in men (beta = 0.063, p = 0.013) but not in women (beta = 0.028, p = 0.242). We were able to observe sex-specific differences in confounding variables, like occupation which was only significant in women, but not in men.

**Table 2:** Overview of models used in the following analysis. 1 – 6 = baseline, 1a – 6a = follow-up. Models b – e are used in the sensitive analysis.

Model Nr	Model description
1, 1a	WHtR ~ CTS sum
2, 2a	WHtR ~ CTS sum + age + educational years + occupation + current depression
3–6,	
3a : e–6a : e	WHtR ~ childhood trauma category

**Table 3:** Results from the multivariable regression models with body shape as outcome−baseline. Model 1 uses the CTS score as predictor for body shape (WtHR) only. To compare the strength of the effect, model 2 includes known sociodemographic variables predicting an increased WHtR. Follow-up: Concerning the CTS sum score as predictor of the WHtR in men, the strength of the effect was similar to the baseline. However, in women the effect was reversed. Age (increased) and educational years (decreased) are the strongest predictors for changes in body shape.

Pooled data model	Variables	Women		Men	
		Beta		Beta	
*Model 1*	CTS sum	0.074**		0.133***	
*Model 2*	CTS sum	0.028		0.063*	
	Age	0.333***		0.327***	
	Education years	−0.157***		−0.137***	
	Occuation	−0.092***		0.003	
	Current depression	− 0.002		0.051	
**Follow-up**					
*Model 1a*	WHtR ~ CTS sum	0.056*		0.116***	
*Model 2a*	CTS sum	0.007		0.043*	
	Age	0.321***		0.261***	
	Education years	−0.162***		−0.143***	
	Occupation	− 0.050		− 0.071*	
	Current depression	0.012		0.064*	

^+^p <  = 0.1, *p <  = 0.05, **p <  = 0.01, ***p <  = 0.001. Statistical significant results are highlighted in bold.

Results of the second survey confirmed these findings (Table 4). The CTS sum score had still a significant impact on the WHtR, the socioeconomic variables age and educational years had the strongest impact (largest beta). Occupation and current depression had significant effects in men, but with low effect size, while in women, the effect of occupation was not significant anymore. It has to be noted that the median age was already relatively high at the baseline (median age = 51 years, s. Table 1).

**Table 4:** Secondary analysis, results from the multivariable regression models with body shape as outcome—baseline: Model 3 – model 6 are showing the effect of each childhood trauma category on body shape. We could identify distinct differences between the sexes. In women, both emotional categories are predictors for an increased WHtR, while in men, the physical categories are relevant. Follow-up: Similar to the baseline, the sexes are affected by different categories. The trend was stable with emotional categories being more relevant in women, while physical trauma was more relevant in men.

Pooled data model	Variables	Women		Men	
		Beta		Beta	
*Model 3*	Emotional neglect	0.083***		*0.051+*	
*Model 4*	Physical neglect	0.035		0.134***	
*Model 5*	Emotional abuse	*0.049 +*		0.043	
*Model 6*	Physical abuse	0.024		0.071**	
*Model 7*	Sexual abuse	−0.007		−0.009	
**Followup**					
*Model 3a*	Emotional neglect	0.062*		0.055*	
*Model 4a*	Physical neglect	0.017		0.101***	
*Model 5a*	Emotional abuse	*0.046 +*		0.025	
*Model 6a*	Physical abuse	0.013		0.063*	
*Model 7a*	Sexual abuse	0.000		0.018	

^+^p <  = 0.1, *p <  = 0.05, **p <  = 0.01, ***p <  = 0.001. Statistical significant results are highlighted in bold.

### Secondary analysis: maltreatment categories as predictors of WHtR

Concerning the sex-specific prevalences of the trauma categories, we were able to identify distinct patterns in women and men across the maltreatment categories. Emotional neglect and abuse had stronger impacts on the body shape in women than in men, and physical neglect and abuse had stronger impacts in men. Nevertheless, the strength of the effects of all variables remained low. These sex-specific patterns did not change much over time. In the follow-up, the effects of the emotional categories were more pronounced in women than in men. However, emotional neglect was a significant predictor for WHtR in men in the follow-up. The physical categories were still significant in men only, but varied slightly in effect sizes (Table 4).

### Sensitivity analysis

Results from the weight-stratified approach showed notable changes, compared to the main analysis . In women of the BMI >  = 25 group, physical abuse became the second strongest predictor, almost equal to emotional neglect, predicting an increased WHtR (Table 5). In the BMI >  = 30 group, no trauma category had a significant effect, except sexual abuse in the follow-up, where the direction was reversed—predicting a lower WtHR (Table 6). In men, only small changes could be identified. Overall, physical neglect remained the strongest predictor (largest beta) for an increased WHtR in both groups BMI >  = 25, BMI >  = 30, baseline and follow-up (Table 5 and Table 6). While we could not identify a significant effect of physical abuse in the BMI >  = 25 group in sensitivity analyses, emotional abuse became a significant predictor for an increased WHtR within the follow-up of the BMI =  > 30 group, with a strength similar to physical neglect (beta physical neglect: 0.113, emotional abuse: 0.107; Table 5 and Table 6).

**Table 5:** Results of the multivariable regression models of the sensitivity analysis—baseline. At BMI >  = 25, the effect of childhood trauma categories was more stable in men than in women. Physical neglect remained the main predictor for an increased WHtR in men, in women, emotional neglect remained the strongest predictor for an increased WHtR, however, compared to the main analysis, physical abuse became a significant predictor as well.

Pooled dataBMI > = 25	Model		Women		Men	
			Beta		Beta	
	*Model 3b*	Emotional neglect	0.083*		0.038	
	*Model 4b*	Physical neglect	*0.065+*		0.110***	
	*Model 5b*	Emotional abuse	0.044		0.033	
	*Model 6b*	Physical abuse	0.081*		0.050	
	*Model 7b*	Sexual abuse	−0.003		−0.002	
**Follow-up**						
	*Model 3c*	Emotional neglect	0.069*		*0.054+*	
	*Model 5c*	Emotional abuse	0.024		0.013	
	*Model 6c*	Physical abuse	0.064*		0.033	
	*Model 7c*	Sexual abuse	−0.004		0.022	

^+^p <  = 0.1, *p <  = 0.05, **p <  = 0.01, ***p <  = 0.001. Statistical significant results are highlighted in bold.

**Table 6:** Results of the multivariable regression models of the sensitive analysis for follow-up. In the obese group, the strength of childhood trauma in women became even weaker and less relevant. However, in the follow-up, we could identify sexual abuse as predictor for a lower WHtR. In men, physical neglect remained the most relevant predictor, however, emotional abuse was significant in the baseline as well.

Pooled data BMI > = 30	Model		Women	Men
			Beta	Beta
	*Model 3d*	Emotional neglect	−0.003	0.046
	*Model 4d*	Physical neglect	0.049	0.113*
	*Model 5d*	Emotional abuse	0.054	0.107*
	*Model 6d*	Physical abuse	0.060	0.056
	*Model 7d*	Sexual abuse	−0.014	-0.014
**Follow-up**				
	*Model 3e*	Emotional neglect	−0.064	0.047
	*Model 4e*	Physical neglect	0.033	0.135*
	*Model 5e*	Emotional abuse	0.017	0.013
	*Model 6e*	Physical abuse	0.039	0.016
	*Model 7e*	Sexual abuse	−0.106*	−0.006

^+^p <  = 0.1, *p <  = 0.05, **p <  = 0.01, ***p <  = 0.001. Statistical significant results are highlighted in bold.

## Discussion

The aim of this study was to examine whether childhood maltreatment, including physical and emotional abuse and neglect, predicts an increase in WHtR, indicating body shape, in sex-stratified analyses through the use of population-based samples from two different regions in Germany. As hypothesized, we were able to show the link between child maltreatment and increased WHtR. However, in our findings the strength of the effect (defined as the proportion explaining the variance of WHtR, beta) is weak (beta = 0.074 for women and beta = 0.133 for men). The effect remained statistically significant in men, but not in women, when controlling for socioeconomic variables like occupation and age. Both socioeconomic factors were far more stronger in their effects, as compared to the trauma categories.

Regarding the maltreatment categories neglect and abuse, we observed distinct patterns in women and men. In general, we identified emotional neglect and physical neglect/abuse as variables that are linked to an increased WHtR in both, women and men. Furthermore, we could detect sex specific patterns on the impact of child maltreatment.

The strength of the effect of overall childhood maltreatment on body shape in adult life was very low. When stratifying our data by weight class, the effect was even weaker and less significant. Especially in the BMI >  = 30 group, physical neglect in men was the only stable factor predicting an increased WHtR in baseline and follow-up. Since we stratified all models by sex, our results are somewhat difficult to compare with studies that did not follow that approach. Our results are in contrast to Williamson et al.^34^, who reported an increased relative risk in individuals with overweight or obesity after the experience of physical and verbal abuse; however the authors did not take sex differences into account. Schulze et al.^5^ reported a significant effect of physical neglect in the BMI, which we could reproduce for the WHtR in men. Bentley et al.^8^ reported trends (p < 0.1) for physical abuse in both sexes. Again, something we were able to reproduce in men, not divided into weight groups and in women, within the BMI >  = 25 group only.

Our results are in line with a recent review and meta-analysis, reporting modest associations of adverse childhood experiences and overweight or obesity^17^, but in contrast to previous studies which reported strong correlations of childhood maltreatment and obesity^35,36^, for review see^7,37^. One explanation could be that our data set is population-based and did not exclude healthy people with a higher BMI. For example, in Amianto et al.^36^ the data is based on patients with severe obesity BMI 40.03 ± 8.43. The same can be said about Richardson et al., there the authors found significant results between sexual and physical abuse in severe obesity (BMI >  = 40), too. This argument was already brought up, e.g. by Pederson and Wilson^38^, citing several studies showing only weak associations of child maltreatment and an increase in body shape. Additionally, reviews and meta-analysis include studies that did not identify child maltreatment as predictor for obesity or an increase in BMI, e.g. ~ 20% of all studies in Danese and Tan^37^.

Furthermore, obesity was not our outcome. We were not interested in the change of probabilities if someone obese had experienced childhood maltreatment. Instead, our aim was to show the actual differences in body shape between people with and without experienced childhood maltreatment.

### Strength and limitations

Since our data is population-based, the number of people with severe obesity and experience in child maltreatment are relatively low, compared to a patient based setting. Furthermore, our age-range is rather small with 35 years as lowest and 67 years as maximum age. Stratification by age, which could be useful since age is the strongest predictor for an increase in body shape, was not appropriate because it could not be justified from a biological and/or sociological point of view. One strength of our study is that it combines data from two different regions, so we were able to identify local differences in the prevalence of experienced child maltreatment. Another advantage is that both populations were similar in sex ratio, socio economic and demographic variables.

### Outlook for further studies

Since we had no clinical population, we could not detect a strong correlation between experienced child maltreatment and an increased WHtR in adult life, as it is reported in clinical studies^36^. A possible explanation could be a U-shaped correlation, as it has been already shown for the link between depression and body shape^39^. Roenholt et al.^40^ demonstrated such a correlation, however, they added, that those results were limited to the age class of 24 years and it remained open whether the results are representative for other age classes as well. Unfortunately, we could not look for such a correlation, since in our sample the number of people with a BMI >  = 40 and high CTS sum scores was too low (e.g. CTS sum score >  = 20, n = 9, all women, median BMI = 26.6). We suggest that this question should be investigated in further studies.

## Methods

### Cohort data

Cohort data were selected from the Study of Health in Pomerania (SHIP) and from the Cooperative Health Research in the Augsburg Region study (KORA). The procedures of obtaining the data have been described in detail elsewhere^41,42^. Briefly, the SHIP-study is a population based epidemiological study from the region of Pomerania, the northeast region of Germany. At its baseline (SHIP-0, 1997–2001), 4,308 subjects aged 20–83 years had participated. The first follow-up (SHIP-1) was conducted in 2002–2006 (n = 3,300), followed by SHIP-2 from 2008–2012 (n = 2,333). Concurrently the SHIP-LEGEND study (Life-Events and Gene-Environment Interaction in Depression) took place (2008; n = 2,400) in which specifically data on psychiatric disorders and their psychosocial correlates were collected. Finally, SHIP-3 was conducted from 2014 to 2016 containing 1,718 participants from the baseline study. For the current study, we use the combined data from SHIP2/SHIP-Legend in our baseline, and SHIP-3 as follow-up. KORA is an expansion of the international MONICA (Monitoring of Trends and Determinants of Cardiovascular Disease) project of the region around Augsburg^42^. The KORA Research platform focuses on epidemiological studies in health economics and health care research. The first survey started in 1986 (S1), followed by three cross-sectional surveys in 1989 (S2), 1994 (S3) and 1999 (S4) with approximately 18,000 individuals aged 25–74 years. For the current study, the two follow-ups F4, 2006 and FF4, 2013^43^ from the S4 cohort were used.

### Exclusion criteria

Since we calculated sum-scores for childhood-trauma as well as current depression, we excluded all participants with missing data, e.g. if a participant had one missing item in the Childhood Trauma Questionnaire^33,44^, the sum and therefore, the binary outcome, was not determined. Furthermore, in KORA, participants older than 67 were not asked about childhood experience, therefore, we excluded all people older 67 years of the baseline population.

### Data harmonization and handling

We used DataSHIELD—Data Aggregation through Anonymous Summary-statistics from Harmonised Individual LevEL Databases—to jointly analyze the cohort data. DataSHIELD enables describing and analyzing large scale and complex interactions in epidemiological studies, by combining cohorts from several distinct studies. Usually, sharing the individual level data necessary for many epidemiological analyses raises concerns about privacy, especially in such sensitive topics like drinking habits, social status and diseases, whereas in DataSHIELD only non-disclosive summary statistics are shared across sites and any individual data remains on local servers and thus remains inaccessible for all parties. An additional server coordinates the analyses between all data servers simultaneously, thus receiving, processing and sending back the result for the requested analysis to the local server^31,32^. Methods like generalized linear models give the same results in DataSHIELD as they would when pooling the data on the individual-level, but methods with the potential to discern individual data (e.g. scatter plots) are prohibit in DataSHIELD and outliers as well as the “tails” for each dataset are replaced by mean values, meaning that the functionality is more limited than in the classical case.

### Measures

#### Childhood maltreatment

In SHIP, childhood maltreatment was obtained with the German version of the childhood trauma questionnaire^45^, a 28-item self-reporting questionnaire with excellent internal consistency^46^. In KORA, the shorter Childhood Trauma Screener^33^ with only 5-items was used. In a first step, we reduced the CTQ to the respective CTS questions in the SHIP sample. The internal consistency between the CTQ and the CTS has been described with an overall Cronbachs α of 0.757, with “physical neglect” as the item with the lowest, but still acceptable, consistency^33^. Next, we calculated the sum score from the remaining five CTS questions. Each of the five question is a proxy for one of the following trauma categories; emotional neglect, physical neglect, emotional abuse, physical abuse and sexual abuse.

Each category has five levels of severity, ranging from *never experienced the specific trauma*^1^ to^5^* very often experienced the specific trauma, the sum score thus ranged from 5–25.* Given the low number of participants with frequent trauma experience (s. supplement), we transformed each trauma category into a binary variable. The threshold for the two neglect categories were the subscales “often” and “very often” while for the two abuse items, it started with “occasionally”. Sexual abuse had the strongest threshold, starting with the rating “rare”^47^.

#### Current depression

Since there is a link between depression and obesity^3^, we decided to use current depression as a control variable. For SHIP 2/LEGENDE, the status of current depression was assessed by the Beck Depression Inventory II (BDI-II;^48^), 21-item self-reporting questionnaire. In SHIP-3, as well as KORA (F4, FF4), the Patient Health Questionnaire^49^ (PHQ-9), a-nine-item self-reporting questionnaire was used. To harmonize the results for both tools, we used the threshold between none/mild depressive episode and medium/severe depressive episode, at a sum of 20 for the BDI-II and a sum of 8 for the PHQ-9. In a next step, the variable “current depression” was transformed into a binary variable with “0” for none/mild and “1” for moderate/severe current depressive episode.

#### Employment status

The employment status has four categories related to working hours/week: (1)  >  = 35 h, (2) 15–34 h, (3) < 15 h and (4) unemployed. Since only few men are working less than 35 h per week (Table 1), we decided to transform this variable into a binary format with 0 = unemployed and 1 = employed.

#### Body shape

Body shape was the primary outcome, assessed by the Waist-to-Height-Ratio (WHtR^50,51^, because it is much more sensitive than the BMI^50,52,53^. Like the BMI, the WHtR can be used as a proxy for an unhealthy body shape composition and is, like the BMI, associated with obesity and metabolic syndrome^50^.

We conducted a sensitivity analysis using the BMI, stratifying the data in the following three weight-classes after the guidelines of the WHO for adults: normal weight, BMI >  = 18.5, overweight, BMI >  = 25 and obese, BMI >  = 30.

### Statistical analysis

In a first step, we described the joint data set as well as each cohort of its own (Table 1). In the next step, we used multivariable linear regression models to identify the effect of the CTS sum score on the primary outcome (body shape as assessed by WHtR). Next, we added sociodemographic control variables, age, current depression, educational years and occupation, to the model. In a second step, we used the five binarised maltreatment categories as independent variables to identify their impact on body shape.

Since we were interested in how much of the variance of body shape is explained by childhood maltreatment (sum score as well as the five binary categories), we repeated all regression models with standardized variables in order to calculate beta square. Since both regional samples had a baseline and a follow-up, the same procedure was repeated with the follow-up data.

As a sensitivity analysis, we created three different weight classes for each cohort. At first, we excluded all individuals with low to normal weight (Included: BMI >  = 18.5). The second weight class included all individuals that are overweight (Included: BMI >  = 25). The last weight class consisted of all individuals with a BMI >  = 30.

For all analyses, we identified the impact (beta) and the explanatory part for each variable of the variance of body shape (beta square). Additionally, all analyses were stratified by sex. All analysis were performed with the BMI as well as the WHtR as outcome continuous variable. Since the WHtR is more sensitive and less vulnerable to age and sex^52^, only results from the WHtR are shown. For results with the BMI as outcome variable, please refer to the supplement. Analyses were conducted in DataSHIELD version 4.1.0 and R 3.5.2^54^.

## Information for the use of KORA data

The informed consent given by KORA study participants does not cover data posting in public databases. However, data are available upon request by means of a project agreement from KORA (http://epi.helmholtz-muenchen.de/kora-gen/). Requests should be sent to kora.passt@helmholtz-muenchen.de and are subject to approval by the KORA Board.

## Information for the use of SHIP data

Data availability is open by request by the Institute of Community Medicine, University of Greifswald at the Transferstelle für Daten- und Biomaterialienmanagement:transfer@uni-greifswald.de.

## Supplementary Information

Supplementary Information.

## Data Availability

GESA is a multi-cohort project building on SHIP and KORA, where the data availability is limited to the local storage guidelines. Data access rights must be requested at each cohort and subsequently data access can be granted by the authors of this paper via DataSHIELD.
