# Supplementary material for: The relation between childhood adversity and adult obesity in a population-based study in women and men
Source: Sci Rep. 2021 Jul 7;11:14068. doi: 10.1038/s41598-021-93242-4 (PMC8263764; doi:10.1038/s41598-021-93242-4)
Supplement: Supplementary file 1 — Supplementary Information. [file 41598_2021_93242_MOESM1_ESM.docx]

**The relation between childhood adversity and adult obesity in a population-based study in women and men**

Toni Fleischer†*. Christine Ulke†. Manfred Beutel. Harald Binder. Elmar Brähler. Hamimatunnisa Johar. Seryan Atasoy. Johannes Kruse. Daniëlle Otten. Ana N. Tibubos. Daniela Zöller. Sven Speerforck. Hans J. Grabe. Karl-Heinz Ladwig. Georg Schomerus

† These authors contributed equally to this work

* corresponding Author

Examples of BMI and the WHtR as outcome variable.

TableS1: CTS sum score as predictor

TableS2: Women, all five childhood trauma categories

TableS3: Men, all five childhood trauma categories

**TabS1: Results from the multivariable regression models with body shape as outcome: baseline.**

In comparison: BMI versus Waist to height ratio (WHtR). The latter is much more sensitive than the BMI.

|  | Estimate | low 0.95CI | high 0.95CI | Std. Error | p-value |
| --- | --- | --- | --- | --- | --- |
| BMI ~ CTS sum |  |  |  |  |  |
| women | 0.043 | -0.007 | 0.093 | 0.025 | 0.093 |
| men | 0.093 | 0.040 | 0.146 | 0.027 | **0.001** |
|  |  |  |  |  |  |
| WtHR ~ CTS sum |  |  |  |  |  |
| women | 0.074 | 0.024 | 0.123 | 0.025 | **0.004** |
| men | 0.133 | 0.081 | 0.185 | 0.027 | **< 0.001** |

**TabS2: Results from the multivariable regression models with body shape as outcome: baseline, women:**

In comparison: BMI versus Waist to Height Ratio (WHtR). Likewise the CTS sum score, the WHtR is more sensitive than the BMI.

|  | Estimate | low 0.95 CI | high 0.95 CI | Std. Error | p-value |
| --- | --- | --- | --- | --- | --- |
| BMI ~ emotional neglect |  |  |  |  |  |
|  | 0.058 | 0.007 | 0.108 | 0.026 | **0.026** |
| BMI ~ emotional abuse |  |  |  |  |  |
|  | 0.036 | -0.015 | 0.086 | 0.026 | 0.169 |
| BMI ~ physical neglect |  |  |  |  |  |
|  | 0.016 | -0.034 | 0.067 | 0.026 | 0.526 |
| BMI ~ physical abuse |  |  |  |  |  |
|  | 0.013 | -0.037 | 0.064 | 0.026 | 0.604 |
| BMI ~ sexual abuse |  |  |  |  |  |
|  | -0.007 | -0.057 | 0.044 | 0.026 | 0.795 |
|  |  |  |  |  |  |
| WHtR ~ emotional neglect |  |  |  |  |  |
|  | 0.083 | 0.033 | 0.133 | 0.026 | **0.001** |
| WHtR ~ emotional abuse |  |  |  |  |  |
|  | 0.049 | -0.001 | 0.099 | 0.026 | *0.057* |
| WHtR ~ physical neglect |  |  |  |  |  |
|  | 0.035 | -0.015 | 0.085 | 0.026 | 0.172 |
| WHtR ~ physical abuse |  |  |  |  |  |
|  | 0.024 | -0.027 | 0.074 | 0.026 | 0.356 |
| WtHR ~ sexual abuse |  |  |  |  |  |
|  | -0.007 | -0.057 | 0.043 | 0.026 | 0.775 |

**TabS3: Results from the multivariable regression models with body shape as outcome: baseline, men:**

In comparison: BMI versus Waist to Height Ratio (WHtR). Emotional abuse is the only variable, which was more sensitive in men with the BMI as outcome, compared with the WHtR as outcome.

|  | Estimate | low 0.95CI | high 0.95CI | Std. Error | p-value |
| --- | --- | --- | --- | --- | --- |
| BMI ~ emotional neglect |  |  |  |  |  |
|  | 0.036 | -0.019 | 0.090 | 0.028 | 0.199 |
| BMI ~ emotional abuse |  |  |  |  |  |
|  | 0.049 | -0.005 | 0.104 | 0.028 | *0.074* |
| BMI ~ physical neglect |  |  |  |  |  |
|  | 0.086 | 0.033 | 0.140 | 0.027 | **0.002** |
| BMI ~ physical abuse |  |  |  |  |  |
|  | 0.063 | 0.008 | 0.117 | 0.028 | **0.023** |
| BMI ~ sexual abuse |  |  |  |  |  |
|  | -0.012 | -0.066 | 0.042 | 0.028 | 0.665 |
|  |  |  |  |  |  |
| WHtR ~ emotional neglect |  |  |  |  |  |
|  | 0.051 | -0.003 | 0.104 | 0.027 | *0.064* |
| WHtR ~ emotinal abuse |  |  |  |  |  |
|  | 0.043 | -0.011 | 0.096 | 0.027 | 0.118 |
| WHtR ~ physical neglect |  |  |  |  |  |
|  | 0.134 | 0.081 | 0.187 | 0.027 | **< 0.001** |
| WHtR ~ physical abuse |  |  |  |  |  |
|  | 0.071 | 0.017 | 0.125 | 0.027 | **0.009** |
| WHtR ~ sexual abuse |  |  |  |  |  |
|  | -0.009 | -0.063 | 0.044 | 0.027 | 0.731 |
